# Tafamidis in the Contemporary Management of Transthyretin Cardiac Amyloidosis: A Systematic Review

**DOI:** 10.3390/jcm15155832

**Published:** 2026-07-25

**Authors:** Ameen Nasser, Mateusz Michalczak, Alexandra Malkowski, Wiktoria Małgorzata Zgoda, Jakub Michalczak, Anna Żądło, Tomasz Tokarek

**Affiliations:** 1 Center for Innovative Medical Education, Jagiellonian University Medical College, 30-688 Krakow, Poland; ameen.nasser@alumni.uj.edu.pl (A.N.); mateusz.michalczak@alumni.uj.edu.pl (M.M.); alexandra.malkowski@student.uj.edu.pl (A.M.); wiktoria.zgoda@student.uj.edu.pl (W.M.Z.); jakub.michalczak@student.uj.edu.pl (J.M.); anna.zadlo@uj.edu.pl (A.Ż.); 2 Center for Invasive Cardiology, Electrotherapy and Angiology, 33-300 Nowy Sacz, Poland

**Keywords:** transthyretin amyloid cardiomyopathy, global left ventricular strain, left ventricular ejection fraction, tafamidis, all-cause mortality, NYHA classification score

## Abstract

**Background**: Transthyretin amyloid cardiomyopathy (ATTR-CM) is a progressive infiltrative cardiomyopathy associated with substantial morbidity and mortality. This systematic review evaluates the effects of tafamidis on echocardiographic parameters, cardiac biomarkers, functional outcomes, mortality, and safety in patients with ATTR-CM. **Methods**: This systematic review was conducted in accordance with PRISMA 2020 guidelines and registered in PROSPERO (CRD420261391553). PubMed, Embase, and Web of Science were searched through 20 March 2026. Eligible studies included adults with ATTR-CM treated with tafamidis and reporting, echocardiographic, biomarker, functional, mortality, or safety outcomes. Randomized controlled trials and observational studies were included. Risk of bias was assessed using the Cochrane RoB 2 tool and Newcastle–Ottawa Scale. Due to substantial heterogeneity, a narrative synthesis was performed. **Results**: Seventeen studies were included with a total of 1890 patients. Tafamidis treatment was potentially associated with stabilization of global longitudinal strain, preservation of functional status, and lower all-cause mortality compared with untreated or control cohorts. Biomarker findings, including N-terminal pro-B-type natriuretic peptide (NT-proBNP) and high-sensitivity cardiac troponin T (hs-cTnT), were heterogeneous and did not demonstrate a consistent pattern of improvement. Functional outcomes, including New York Heart Association (NYHA) class, 6-minute walk distance (6MWT), National Amyloidosis Center (NAC) staging, and quality-of-life measures, suggested slower clinical deterioration among treated patients. Limited available safety data indicated that tafamidis was generally well tolerated, with no major safety concerns identified. **Conclusions**: Current evidence may suggest that tafamidis slows disease progression and may potentially be associated with improved survival in ATTR-CM. Further prospective studies with standardized outcome reporting are needed. Evidence was limited by heterogeneity in study design, outcome reporting, and follow-up duration, with most included studies being observational.

## 1. Introduction

Cardiac amyloidosis (CA) is a complex and heterogeneous infiltrative cardiomyopathy characterized by the extracellular deposition of insoluble misfolded protein fibrils within the myocardium, leading to progressive structural and functional impairment of the heart [1]. This pathological process disrupts myocardial architecture, resulting in increased ventricular wall thickness, reduced compliance, and ultimately a restrictive cardiomyopathy phenotype associated with heart failure, arrhythmias, and conduction abnormalities [1,2].

The two principal subtypes of cardiac amyloidosis are transthyretin amyloidosis (ATTR) and light-chain cardiac amyloidosis (AL-CA), which together account for more than 95% of all cases [2]. Although once considered a rare disease, CA is increasingly recognized as a significant and underdiagnosed contributor to cardiovascular morbidity, particularly in older populations. Contemporary data suggest that up to 15–16% of patients with heart failure—especially those with preserved ejection fraction (HFpEF)—may have underlying ATTR that remains undiagnosed [2,3]. Furthermore, specific populations, such as elderly males and individuals with certain genetic variants, are at increased risk, underscoring the importance of targeted screening strategies [1].

The clinical importance of cardiac amyloidosis lies in its progressive and often fatal nature if untreated. Amyloid fibril deposition leads to diastolic dysfunction, restrictive filling patterns, and eventual systolic impairment, accompanied by a high burden of arrhythmias such as atrial fibrillation and conduction system disease. These complications contribute to frequent hospitalizations, reduced quality of life, and increased mortality [1,3].

Tafamidis has emerged as a pivotal therapeutic agent for ATTR cardiomyopathy (ATTR-CM). Tafamidis acts by stabilizing the transthyretin tetramer, thereby preventing its dissociation into monomers [2]. Clinical trials, particularly the ATTR-ACT trial by Maurer et al. [4], have demonstrated that tafamidis significantly reduces all-cause mortality and cardiovascular-related hospitalizations while improving functional capacity and quality of life [2,4]. Real-world evidence further supports its role in reducing amyloid deposition and slowing disease progression, establishing it as the only pharmacologic therapy for ATTR-CM approved by both the U.S. Food and Drug Administration and the European Commission [2,5].

Despite these therapeutic advances, awareness of cardiac amyloidosis remains suboptimal among clinicians, contributing to underdiagnosis and delayed treatment initiation. Increasing awareness, improving access to non-invasive diagnostic tools such as nuclear scintigraphy and cardiac magnetic resonance imaging, and fostering multidisciplinary collaboration are essential steps toward optimizing patient outcomes [1,2].

In light of these considerations, the purpose of this systematic review is to provide a comprehensive evaluation of the clinical outcomes associated with tafamidis therapy in patients with ATTR-CM.

## 2. Materials and Methods

### 2.1. Study Protocol

We performed an initial search encompassing our research question (see Table 1). A protocol was then produced following the PRISMA 2020 statement: an updated guideline for reporting systematic reviews (Page, 2021) [6]. As Supplementary Materials, readers can refer to the PRISMA checklist.

### 2.2. Search Strategy

Three independent authors conducted the literature search on the 20 March 2026. The databases searched included PubMed, Embase, and Web of Science. The search strategy was developed using two main search lines: tafamidis and ATTR-CM. The secondary search line consisted of synonymous terms describing ATTR-CM, including ‘*transthyretin cardiomyopathy*’, ‘*ATTR cardiomyopathy*’, and ‘*cardiac amyloidosis*’, to maximize search sensitivity. The overall search strategy was applied consistently across all databases and adapted only to accommodate the specific indexing system and search syntax of each database. The following search strategy was used:

(1) Pubmed: (*“Tafamidis”[Mesh] OR tafamidis[tiab]) AND (“Amyloidosis”[Mesh] OR “transthyretin cardiac amyloidosis”[tiab] OR “transthyretin cardiomyopathy”[tiab] OR “ATTR cardiomyopathy”[tiab] OR “cardiac amyloidosis”[tiab]).*
(2) Embase: *(‘tafamidis’/exp OR tafamidis:ti,ab) AND (‘amyloidosis’/exp OR ‘transthyretin cardiomyopathy’:ti,ab OR ‘transthyretin cardiac amyloidosis’:ti,ab OR ‘cardiac amyloidosis’:ti,ab OR ‘ATTR cardiomyopathy’:ti,ab).*
(3) Web of Science *TS = ((tafamidis) AND (“amyloidosis” OR “transthyretin cardiac amyloidosis”OR “transthyretin cardiomyopathy” OR “cardiac amyloidosis” OR “ATTR cardiomyopathy”).*

The study was reported in accordance with the PRISMA guidelines and registered with PROSPERO; ID number CRD420261391553.

### 2.3. Study Selection and Eligibility Criteria

**Figure 1:**
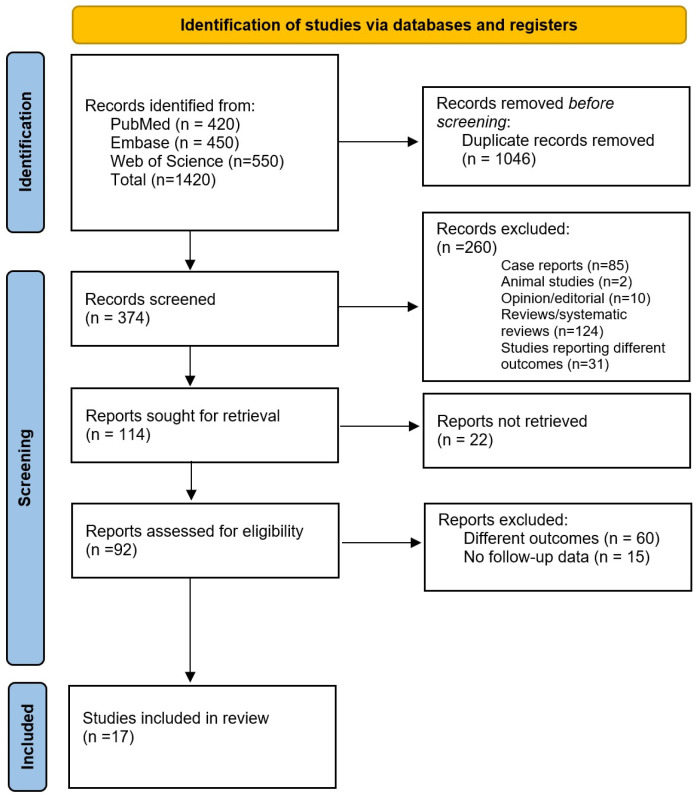
PRISMA flow diagram illustrating the study selection process for this systematic review.

Following completion of the literature search, all retrieved studies were imported into Zotero for reference management, and duplicate records were removed (see Figure 1). Three independent reviewers subsequently screened the studies based on titles and abstracts. Studies were eligible if they included adults with ATTR-CM treated with tafamidis and reported at least one prespecified clinical, echocardiographic, biomarker, functional, mortality, or safety outcome. Randomized controlled trials (RCTs), prospective cohort studies, retrospective cohort studies and case–control studies were eligible for inclusion. Case reports, animal studies, editorials/opinion articles, conference abstracts, reviews, non-English studies, studies unrelated to cardiac amyloidosis, and studies not reporting outcomes of interest were excluded.

The same reviewers then independently performed full-text screening of the eligible articles. Upon completion of the full-text review, the reviewers exchanged the articles and repeated the screening process to minimize selection bias. Following this step, consensus was reached among the reviewers regarding the final list of studies to be included in the systematic review.

### 2.4. Data Extraction

Five independent reviewers extracted data from studies that met the inclusion criteria for the systematic review. Data were collected for both patients with ATTR and the control group whenever present and available. The following variables were extracted: (1) study name and year of publication; (2) study design; (3) sample size; (4) median age and age range of participants; (5) number of patients with wild-type ATTR (ATTRwt) and variant/hereditary ATTR (ATTRv); (6) sex distribution; (7) global longitudinal strain (GLS); (8) left ventricular ejection fraction (LVEF); (9) N-terminal pro–B-type natriuretic peptide (NT-proBNP) levels; (10) high-sensitivity cardiac troponin T (hs-cTnT) levels; (11) National Amyloidosis Centre (NAC) score; (12) 6-minute walk test (6MWT); (13) Kansas City Cardiomyopathy Questionnaire (KCCQ) score; (14) New York Heart Association (NYHA) functional classification; (15) all-cause mortality; (16) safety profile; and (17) duration of follow-up.

For each extracted outcome, both baseline and follow-up values were collected. Data extraction was performed independently by the five reviewers. Any disagreements or conflicts arising during the extraction process were resolved through discussion and consensus with the other authors.

### 2.5. Quality Assessment

The methodological quality of the included non-randomized studies was assessed using the Newcastle–Ottawa Scale (NOS) [7]. Risk of bias in non-randomized studies was evaluated under the following domains: representativeness of the exposed cohort, selection of the non-exposed cohort, ascertainment of exposure, demonstration that the outcome of interest was not present at baseline, comparability based on age and sex, comparability based on additional factors, outcome assessment, adequacy of follow-up duration, adequacy of follow-up completeness, and overall risk of bias. The extracted quality assessment data were subsequently uploaded to CritiPlot [8] to generate a traffic-light plot.

For RCTs, the Cochrane Risk of Bias 2 Tool (RoB2) [9] was used to evaluate risk of bias across the following domains: random sequence generation (selection bias), allocation concealment (selection bias), blinding of participants and personnel (performance bias), blinding of outcome assessment (detection bias), incomplete outcome data (attrition bias), selective outcome reporting (reporting bias), and other potential sources of bias. The findings were represented in a Risk of Bias 2 diagram using the RoB 2 visualization tool [9].

Five independent reviewers assessed the risk of bias in the included studies. Any discrepancies were resolved through discussion and collaboration, and a final consensus-based risk-of-bias assessment was produced.

Two independent reviewers assessed the certainty of the results using the Grading of Recommendations, Assessment, Development, and Evaluations (GRADE) framework. Outcomes were rated as high, moderate, low, or very low certainty based on study limitations, inconsistency, indirectness, imprecision, and potential publication bias. Randomized controlled trial evidence was initially considered high certainty, while observational studies were considered low certainty and could be upgraded or downgraded depending on GRADE criteria. The overall certainty rating for each outcome was determined through qualitative synthesis of the available evidence across included studies in terms of study type and outcome reporting methods.

### 2.6. Data Synthesis

Due to substantial clinical and methodological heterogeneity among the included studies, quantitative meta-analysis was not performed. Sources of inconsistency included variation in study design, follow-up duration, outcome definitions, reporting formats, ATTR subtype distribution, and availability of comparator groups. Therefore, findings were synthesized using a structured narrative synthesis approach. Outcomes were grouped into echocardiographic, biomarker, functional, mortality, and safety domains, and findings were summarized according to the direction, consistency and generalized patterns of reported effects.

## 3. Results

### 3.1. General Characteristics

Cohort sizes varied substantially, ranging from small studies such as Chamling et al. [10], *n* = 40, and Eguchi et al. [11], *n* = 27, to large trials such as Maurer et al. [4], *n* = 441 (see Table 2). The majority of patients were classified as ATTRwt across studies, with consistently high proportions in most cohorts, including those reported by Rettl et al. [12], Takashio et al. [13], and Zlibut et al. [14]. Variant or hereditary ATTR cases were less frequently represented overall, though they constituted a substantial proportion in selected studies such as Shin et al. [15] and Maurer et al. [4]. Several studies reported exclusively ATTRwt populations, including Chamling et al. [10], Kuyama et al. [16], and Ney et al. [17], highlighting a predominance of wild-type disease representation across the evidence base. The study by Kuyama et al. [18] did not provide subtype classification, limiting detailed subgroup interpretation. In summary, the dataset is characterized by marked heterogeneity in sample size and a clear predominance of ATTRwt cohorts, with relatively limited representation of variant ATTR across most included studies.

Because the included studies comprised randomized trial evidence, controlled observational studies, and uncontrolled before and after studies, their findings were interpreted according to the level of causal inference supported by each design. Randomized trial data were considered the strongest evidence for treatment effects, whereas controlled observational studies were interpreted as associations and uncontrolled before–after studies were considered supportive only for longitudinal trends after tafamidis initiation.

**Figure 2:**
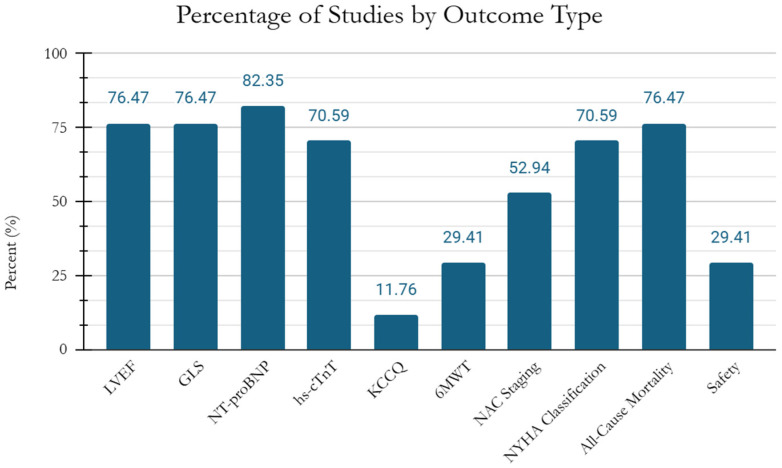
Percentage distribution of reported outcomes across included studies.

The included studies reported a range of clinical, imaging, biomarker, mortality, and safety outcomes (Table 3). LVEF and GLS were each reported in 13 studies, making them the most consistently used imaging endpoints. NT-proBNP was assessed in 14 studies, the most frequently reported biomarker, while hs-cTnT was included in 12 studies. NYHA class was reported in 12 studies, and NAC in eight studies. All-cause mortality was included in 13 studies, although not uniformly defined or consistently prioritized across studies. Functional capacity outcomes were less commonly assessed, with the 6MWT reported in five studies and KCCQ in only two studies. Safety outcomes were reported in five studies (see Figure 2). Taken together, reporting on outcomes was balanced to a certain extent across the studies with the exception of the KCCQ score.

### 3.2. Echocardiographic Outcomes

Across the included studies, GLS findings suggested that tafamidis may have been associated with stabilization of myocardial deformation parameters or a slower rate of decline compared with untreated cohorts. While substantial improvement in GLS was uncommon, the majority of studies demonstrated preservation of strain measurements over time, supporting the proposed role of tafamidis in slowing disease progression rather than reversing established myocardial damage. For example, in the studies by Kuyama et al. [16] and Takashio et al. [13], GLS remained essentially unchanged during follow-up, shifting from −8.4% to −8.5% (*p*-value 0.21) and from −8.3% to −8.4% (*p*-value 0.33), respectively. Similarly, Palmiero et al. [21] reported stable GLS values (−10.8% to −10.9%) (*p*-value 0.873), while Ney et al. [17] observed a slight improvement from −11.0% to −11.5% with a *p*-value of 0.877. Although modest worsening was noted in some tafamidis-treated cohorts, such as the studies by Chamling et al. [10] and Rettl et al. [22], the overall pattern across studies supports the role of tafamidis in slowing deterioration and myocardial deformation rather than restoring normal cardiac mechanics.

The GLS stabilization pattern was particularly evident in studies that included untreated comparator groups, where greater deterioration in GLS was generally observed during follow-up. In the untreated cohort reported by Shin et al. [15], GLS worsened substantially from −12.6% to −9.7%, while the tafamidis-treated group in the same study showed a smaller decline from −13.9% to −12.9%. The *p*-values associated with change in GLS in the study (*p*-value 0.002) were associated with statistical significance in patients who were treated with 20 mg of tafamidis. Likewise, the untreated cohort in the study by Rettl et al. [12] showed deterioration from −11.71% to −10.59% (*p*-value 0.001), compared with relative stability in treated patients. Similar trends were observed in the untreated subgroup analyzed by Takashio et al. [13], where GLS worsened from −9.8% to −9.3% (*p*-value 0.33).

Similarly, the included studies that reported LVEF demonstrated a stabilizing pattern rather than a consistent improvement. In several studies, control groups showed a significant decline in LVEF compared with the treatment groups. However, the overall trend across the included studies suggests that LVEF was generally preserved or only mildly reduced depending on the follow-up time of each study. It is worth noting that LVEF is not a reliable measure of disease progression or severity. The *p*-values in most studies suggest that LVEF results did not reach statistical significance. ATTR-CM can be severe and progress with preserved LVEF.

### 3.3. Cardiac Biomarkers

**Figure 3:**
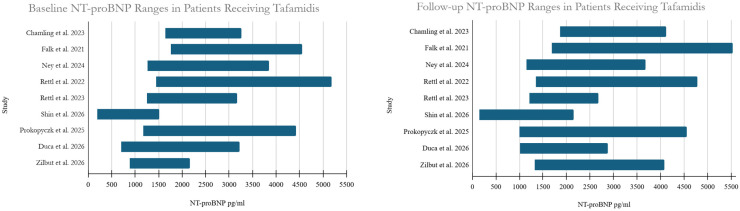
Baseline NT-proBNP concentrations in patients receiving tafamidis, as well as follow-up NT-proBNP levels measured after variable durations of treatment. (Follow-up timepoints differed substantially across the included studies and data was retrieved from Data are derived from Chamling et al. [10], Falk et al. [20], Ney et al. [17], Rettl et al. [12,22], Shin et al. [15], Gawor-Prokopczyk et al. [24], Duca et al. [25], and Zlibut et al. [14]).

Most studies did not demonstrate statistically significant reductions in NT-proBNP during follow-up, and considerable heterogeneity was observed in both reporting methods and biomarker trajectories. Nevertheless, studies that included untreated comparator groups generally reported higher baseline and follow-up NT-proBNP levels, as well as greater increases over time, suggesting that tafamidis may attenuate biomarker progression in some patients despite the absence of a consistent reduction in circulating natriuretic peptide concentrations. Among the nine of fourteen studies that reported NT-proBNP changes using comparable reporting formats (see Figure 3), a broadly consistent pattern of NT-proBNP ranges was observed. Takashio et al. [13] and Kuyama et al. [16], which reported BNP rather than NT-proBNP, likewise found no significant biomarker changes during follow-up. Although some studies reported reductions in the overall range of NT-proBNP values, these findings were not always reflected in measures of central tendency. For example, Duca et al. [25] observed a reduction in the reported range despite an increase in median NT-proBNP levels (*p*-value 0.493).

In addition to that, hs-cTnT concentrations in patients with ATTR-CM treated with tafamidis were generally stable over time, with no consistent evidence of a uniform decline in biomarker levels. Furthermore, baseline hs-cTnT values varied substantially across cohorts and assay types, ranging from low-sensitivity measurements reported in ng/mL to high-sensitivity assays reported in ng/L, reflecting heterogeneity in laboratory methods and study populations. In several comparative studies, tafamidis-treated groups demonstrated small absolute changes in hs-cTnT levels, with some cohorts showing modest reductions and others showing modest increases over follow-up, like those in Falk et al. [20] (*p*-value 0.057), Kuyama et al. [18] (*p*-value < 0.01), Takashio et al. [13] (*p*-value 0.03), and Ney et al. [17].

In contrast, untreated comparator groups more frequently exhibited higher baseline hs-cTnT concentrations and a tendency toward greater increases over time, as observed in subsets of the Shahi et al. [23], Rettl et al. [12], and Kuyama et al. [18] cohorts. However, this pattern was not uniform across all studies. Studies reporting longitudinal or subgroup analyses like Dobner et al. [19] and Gawor-Prokopczyk et al. [24] similarly demonstrated heterogeneous trajectories, with both stability and mild progression observed depending on cohort definition and access to therapy.

### 3.4. Functional Outcomes

Across the 17 included studies, functional outcomes were heterogeneously reported. NYHA functional class was the most frequently reported functional measure, whereas 6MWT and KCCQ were reported less consistently. NAC stage was reported in approximately half of the studies and was most commonly used to characterize baseline disease severity rather than as a consistent longitudinal functional outcome. Where NAC stage or related biomarker-based disease-severity measures were reported, more advanced disease stage or progression in staging markers was associated with worse clinical outcomes. The functional outcomes suggest that the principal clinical effect of tafamidis is the preservation of functional status rather than substantial improvement in established disease manifestations. The overall evidence indicates a trend toward slower functional decline among tafamidis-treated patients as reported in Palmiero et al. [21], Shin et al. [15], and Kuyama et al. [18]. NYHA class was reported in the majority of studies, although reporting varied substantially, including baseline class distribution, follow-up distribution, median NYHA class, and dichotomized NYHA categories. Among studies with longitudinal NYHA data, tafamidis-treated patients generally demonstrated stable or less worsening functional class compared with untreated or control groups. Objective functional and quality-of-life measures were less frequently available. The 6MWT was reported in a minority of studies. In Maurer et al. [4], tafamidis significantly reduced the decline in 6MWT distance compared with placebo over 30 months. KCCQ was reported in only a small number of studies. In Maurer et al. [4], tafamidis reduced the decline in the KCCQ overall summary score compared with placebo, while Palmiero et al. [21] reported improvement in KCCQ from baseline to 12 months after tafamidis treatment.

### 3.5. Mortality

Mortality and hospitalization outcomes were reported more consistently than functional outcomes, although hospitalization definitions varied across studies (see Table 4). All-cause mortality or a mortality-related endpoint generally favored tafamidis treatment. Despite differences in study design, patient populations, and follow-up duration, the majority of investigations demonstrated a favorable association between tafamidis treatment and survival. Compared with untreated or control cohorts, patients receiving tafamidis generally experienced lower mortality rates (*p*-value < 0.001), suggesting a potential association in which transthyretin stabilization may translate into improved long-term clinical outcomes beyond the preservation of cardiac structure and function. In Maurer et al. [4], tafamidis reduced all-cause mortality compared with placebo, while observational cohorts such as Shahi et al. [23] and Shin et al. [15] also reported lower mortality among tafamidis-treated patients compared with untreated groups, with *p*-values of 0.11 and <0.001, respectively. In Gawor-Prokopczyk et al. [24], disease-modifying treatment, predominantly tafamidis, was associated with a lower rate of death or heart transplantation compared with no disease-modifying therapy (*p*-value 0.005). Cardiovascular or heart failure hospitalization outcomes were less consistent. Some studies reported cardiovascular-related hospitalization, others reported heart failure hospitalization, and others used broader outcomes such as worsening heart failure, which included hospitalization or urgent outpatient visits. Overall, mortality outcomes showed the most consistent benefit with tafamidis, while cardiovascular or heart failure hospitalization outcomes were more heterogeneous, likely reflecting differences in outcome definitions, follow-up duration, baseline disease severity, and study design.

### 3.6. Safety

Safety data were available from a limited subset of studies (5 out of 17) but provided a broadly consistent assessment of tafamidis tolerability. Although adverse event reporting was not standardized across the literature, the available evidence did not identify major safety concerns or unexpected treatment-related complications. Overall, the findings suggest that tafamidis is generally well tolerated in patients with ATTR-CM, supporting its favorable tolerability as a long-term disease-modifying therapy in routine clinical practice. In Maurer et al. [4], tafamidis demonstrated a similar safety profile to placebo. The remaining studies either provided limited safety details or did not report safety outcomes in a format that allowed consistent comparison across cohorts. Collectively, available evidence suggests a favorable safety profile, although conclusions are limited by sparse reporting. One study (Takashio et al. [13]) reported that the dose of tafamidis had to be reduced due to gastrointestinal symptoms in one patient.

### 3.7. Risk-of-Bias Assessment

**Figure 4:**
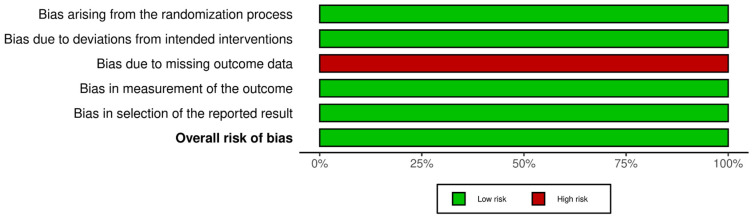
RoB 2 assessment of the ATTR-ACT trial by Maurer et al. (2018) [4], illustrating domain-specific and overall risk-of-bias judgments. (Figure created using Robvis [9]).

Risk-of-bias was assessed for the ATTR-ACT RCT conducted by Maurer et al. [4] using the RoB 2 tool. The tool demonstrated overall low methodological risk. Randomization, blinding, outcome measurement and reporting domains were assessed as low risk. However, bias due to missing outcome data was judged high risk because of substantial and differential attrition over the 30-month follow-up period in this progressive and fatal disease population (see Figure 4).

**Figure 5:**
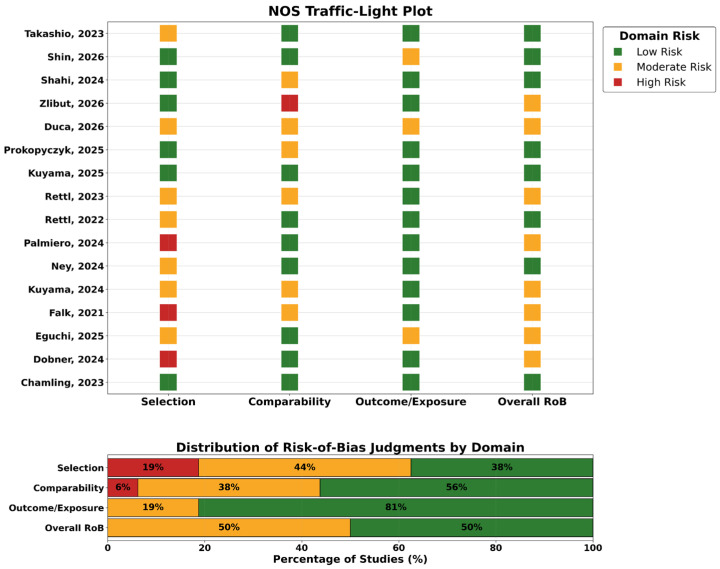
Traffic-light plot summarizing the risk-of-bias assessment across the observational studies included in this systematic review. (Figure made using CritiPlot [8] and data retrieved from Data are derived from Chamling et al. [10], Falk et al. [20], Ney et al. [17], Rettl et al. [12], Shin et al. [15], Gawor-Prokopczyk et al. [24], Duca et al. [25], Zlibut et al. [14], Takashio et al. [13], Dobner et al. [19], Eguchi et al. [11], Kuyama et al. [16], Palmiero et al. [21], Retll et al. [22], Kuyama et al. [18], Shahi et al. [23]).

Most studies demonstrated low risk of bias in the Outcome/Exposure domain, with 81% of studies rated as low risk and no studies classified as high risk in this category (see Figure 5). The Comparability domain showed generally favorable methodological quality, with 56% of studies rated as low risk, although 38% were considered moderate risk and 6% high risk due to limited adjustment for confounding variables.

Among the non-randomized studies, the Selection domain exhibited the greatest variability in study quality, with only 38% of studies assessed as low risk, while 44% were rated moderate risk and 19% high risk, reflecting concerns related to cohort selection, representativeness, or ascertainment methods. Most observational studies were susceptible to treatment selection bias because the tafamidis-treated patients often differed from untreated cohorts based on disease stage and treatment eligibility. Furthermore, immortal time bias may have favored the treated cohorts because patients must survive long enough to receive tafamidis therapy and be available for outcome reporting.

Overall, 50% of studies were judged to have low overall risk of bias, and 50% demonstrated moderate overall risk. No studies were classified as having high overall risk of bias. Collectively, these findings suggest that the included observational studies were generally of moderate to good methodological quality, with the primary limitations arising from selection methods and comparability between study groups.

### 3.8. Certainty Assessment

The certainty of evidence varied across the assessed outcomes according to the GRADE framework (see Table 5). Evidence supporting the beneficial effect of tafamidis on all-cause mortality was downgraded one level and judged to be of moderate certainty, reflecting the consistency of findings across both the ATTR-ACT RCT by Maurer et al. [4] and multiple observational studies, despite some concerns regarding heterogeneity and risk of bias. Evidence for functional outcomes, including NAC staging, NYHA class, 6MWT, and KCCQ score, was considered of low to moderate certainty because of variability in outcome reporting, differences in follow-up duration, and the predominance of observational data. Echocardiographic outcomes, including GLS and LVEF, were rated as low certainty due to reliance on observational studies and heterogeneity in imaging assessment and reporting. Similarly, evidence for biomarker outcomes, including NT-proBNP and hs-cTnT, was judged to be of low certainty because of inconsistent findings across studies and substantial variability in measurement and reporting methods. Safety outcomes were also considered low certainty, primarily due to limited reporting and the relatively small number of studies that systematically assessed adverse events. Across studies, the certainty of evidence was highest for mortality outcomes and lower for imaging, biomarker, and safety endpoints, highlighting the need for additional prospective studies with standardized outcome reporting.

The main reasons for downgrading certainty differed by outcome domain. Mortality was downgraded mainly because, although supported by randomized evidence, the overall synthesis also included heterogeneous observational studies with potential selection bias and limited comparability. Echocardiographic outcomes were downgraded for study limitations, inconsistency in reporting, and reliance mainly on observational or before–after data. Biomarker outcomes were downgraded because NT-proBNP and hs-cTnT results were inconsistent across studies and were reported using heterogeneous measurement units, timepoints, and summary statistics. Functional outcomes were rated low to moderate because randomized data were available for 6MWT and KCCQ, whereas NYHA and NAC findings were mostly derived from observational studies with variable reporting. Safety outcomes were downgraded because adverse events were reported in only a limited subset of studies and were not assessed using standardized methods. No outcome domain was upgraded because the evidence did not consistently meet upgrading criteria such as a large magnitude of effect, clear dose–response gradient, or sufficient control of residual confounding.

## 4. Discussion

The present review highlights a heterogeneous evidence base in ATTR-CM, with marked variation in cohort size, subtype representation, and outcome reporting. The predominance of ATTRwt likely reflects its higher prevalence in older heart failure populations, whereas the under-representation of variant ATTR limits generalizability [26]. Despite this variability, consistent reporting of NT-proBNP, LVEF, and GLS supports their role as core markers of disease severity and longitudinal monitoring. These findings align with evidence from tafamidis trials and prior reviews demonstrating improved survival, reduced hospitalization, and slower functional decline, particularly in ATTRwt populations [4].

Throughout the included studies, GLS may have greater sensitivity to disease severity than LVEF. This aligns with its proposed pathophysiological basis, as amyloid deposition predominantly involves the subendocardial layers, leading to early impairment of longitudinal myocardial contraction. In contrast, LVEF may remain preserved even in advanced disease due to compensatory mechanisms, including increased wall thickness and maintained radial contraction, which help preserve stroke volume despite progressive myocardial infiltration. Overall, these findings suggest that GLS provides a more sensitive and integrated assessment of disease severity in ATTR-CM than LVEF alone.

Across the included studies, tafamidis treatment does not appear to produce consistent reductions in NT-proBNP or hs-cTnT levels. This aligns with the proposed mechanism of tafamidis as a transthyretin stabilizer that reduces further amyloid deposition rather than reversing established myocardial infiltration [27]. However, in this review direct comparison of hs-cTnT levels between studies was limited by assay heterogeneity and different reporting variables. Prior evidence from pivotal trials and pooled analyses similarly reports minimal changes in natriuretic peptides despite clear clinical benefits, including reduced mortality and hospitalization rates and preservation of functional capacity in ATTR-CM populations [4,26]. Importantly, the absence of consistent improvement in NT-proBNP and hs-cTnT should be regarded as a central finding of this review. Therefore, broader claims of clinical benefit should be interpreted as reflecting possible stabilization of disease progression and survival benefit, rather than consistent biochemical improvement.

Despite heterogeneous biomarker findings, functional, mortality, and safety outcomes may suggest the clinical benefit of tafamidis in ATTR-CM, although this benefit appears to be reflected more clearly by functional stabilization and survival outcomes than by consistent improvement in NT-proBNP or hs-cTnT. This lack of consistent biomarker improvement represents an important finding and suggests that NT-proBNP and hs-cTnT changes should not be used alone to define response to tafamidis.

The synthesis of functional, mortality, and safety outcomes suggests more clinical benefits of tafamidis in ATTR-CM than are apparent from biomarker-based measures alone, despite substantial heterogeneity in outcome reporting. Functional measures such as NYHA class may have favored stabilization or slower deterioration in tafamidis-treated patients, while the more limited data from 6MWT and KCCQ assessments similarly reflected preservation of exercise capacity and quality of life, consistent with broader observational evidence of functional benefit in ATTR-CM populations. Mortality outcomes demonstrated the most consistent signal, with both clinical trial and real-world data indicating reduced all-cause mortality and improved transplant-free survival among patients receiving tafamidis, as supported by integrated analyses of disease-modifying therapy in ATTR-CM [26]. In contrast, safety reporting was limited but generally reassuring, with no new safety concerns identified and a favorable tolerability profile consistent with broader reviews of transthyretin stabilizer therapy. These findings reinforce tafamidis as a therapy that stabilizes functional decline and improves survival, while underscoring the need for more standardized reporting of functional and safety outcomes in future ATTR-CM research. However, these findings should be interpreted in light of the methodological limitations identified in the risk-of-bias assessment. In particular, selection bias, limited comparability between treated and untreated cohorts, and possible immortal time bias may have favored tafamidis-treated groups in observational studies. Therefore, while randomized trial evidence supports a treatment effect of tafamidis, findings from non-randomized studies should be interpreted primarily as associations rather than definitive causal effects.

Mortality was the most consistently reported and clinically important endpoint in this review. In the ATTR-ACT randomized trial, tafamidis demonstrated a mortality benefit compared with placebo, providing the strongest causal evidence for improved survival. Observational cohorts were generally directionally consistent with this finding, reporting lower mortality or improved transplant-free survival among patients receiving tafamidis compared with untreated groups. However, unlike ATTR-ACT, these real-world studies were more susceptible to selection bias, limited comparability between treatment groups, and immortal time bias, meaning that their mortality findings should be interpreted as supportive associations rather than definitive causal evidence.

The risk-of-bias assessment (Section 3.7) demonstrated the greatest variability in the selection domain of the NOS, largely driven by the inclusion of small-cohort and single-center observational studies, which may have introduced selection bias and limited generalizability. In contrast, the randomized ATTR-ACT trial assessed using the RoB 2 tool demonstrated an overall low risk of bias across most domains; however, some concerns were noted regarding incomplete outcome data. Specifically, approximately 11% of participants did not complete the full 30-month follow-up, with missing outcome data due to withdrawal, loss to follow-up, or death prior to scheduled assessments, which may have introduced a degree of attrition bias. Overall, while randomized evidence was methodologically robust, observational studies showed greater variability in bias risk, particularly related to study design and participant selection.

### 4.1. Study Limitations

This study is limited by substantial heterogeneity in study design, outcome definitions, follow-up duration, and reporting methods across the included studies. Although some studies enrolled relatively large cohorts, longitudinal analyses were frequently restricted by incomplete baseline or follow-up data, potentially introducing selection bias. Furthermore, the majority of included studies were observational, with only one randomized controlled trial available, limiting the strength of causal inference. In addition, variability in the assessment and reporting of functional, echocardiographic, biomarker, and hospitalization outcomes limited direct comparison across studies. Finally, the heterogeneity of the available evidence precluded formal quantitative synthesis, and therefore the findings should be interpreted primarily as overall trends rather than precise estimates of treatment effect. Although a structured narrative synthesis was used, this approach remains less precise than quantitative meta-analysis and may be more susceptible to subjective interpretation, particularly because studies differed in design, comparator availability, follow-up duration, outcome definitions, and reporting methods. In addition to that, Eguchi et al. [11] reported an inconsistency in which the total sample size was 20 patients, whereas the reported NYHA class distribution accounted for 21 patients, suggesting either a typographical error or a discrepancy in data reporting that could not be resolved from the published manuscript. Dosing regimens also varied across studies depending on the needs of the patients. Therefore, the findings should be interpreted with caution, with greater emphasis placed on overall trends rather than precise between-study comparisons or causal inferences.

The majority of the included studies comprised patients with ATTRwt, with limited representation of ATTRv. This imbalance may limit the generalizability of the findings to the broader ATTR-CM population. ATTRwt and ATTRv differ in terms of genetic background, age of onset, and disease progression, which may also influence clinical presentation and treatment response. The predominance of ATTRwt in the literature may be attributed to its higher prevalence in older populations. Another plausible explanation is that ATTRwt is more frequently diagnosed in contemporary clinical practice due to increased awareness and advances in non-invasive diagnostic techniques, whereas ATTRv remains relatively rarer and more geographically clustered. Therefore, caution is warranted when extrapolating these results to patients with ATTRv. Further studies specifically including larger ATTRv cohorts are needed to better define the efficacy and cardiac effects of tafamidis in this subgroup.

### 4.2. Clinical Implications

The findings suggest that clinicians should not rely exclusively on changes in NT-proBNP or hs-cTnT when assessing response to tafamidis. Preservation of GLS, functional status, and survival may represent more clinically meaningful indicators of treatment benefit. Early diagnosis and initiation of therapy remain critical because tafamidis appears to slow progression rather than reverse established myocardial damage.

### 4.3. Future Directions

In addition to transthyretin stabilizers such as tafamidis, several emerging disease-modifying therapies are likely to further transform the management of ATTR-CM. These therapies target different stages of the amyloid cascade and may ultimately expand treatment options beyond transthyretin stabilization alone. As summarized by Dimza et al. [28], RNA-silencing therapies, including vutrisiran and eplontersen, reduce hepatic transthyretin production, whereas acoramidis is a next-generation transthyretin stabilizer designed to achieve near-complete stabilization of the transthyretin tetramer.

Acoramidis has emerged as a promising alternative transthyretin stabilizer, demonstrating significant improvements in mortality, cardiovascular hospitalizations, functional capacity, and cardiac biomarkers. Owing to its enhanced transthyretin-stabilizing properties, acoramidis may represent an important therapeutic option alongside tafamidis. However, the absence of direct head-to-head trials comparing acoramidis with tafamidis limits definitive conclusions regarding their relative efficacy. Furthermore, differences in study design, patient populations, disease severity, and outcome definitions make indirect comparisons challenging. Consequently, tafamidis and acoramidis should currently be viewed as complementary therapeutic options rather than competing agents until comparative effectiveness studies become available [28,29].

RNA-silencing therapies offer a fundamentally different therapeutic approach by reducing hepatic transthyretin synthesis instead of stabilizing circulating transthyretin. As reviewed by Dimza et al. [28], vutrisiran has demonstrated favorable effects on cardiovascular outcomes, functional capacity, and quality of life in patients with ATTR-CM, supporting its potential role as either an alternative or adjunctive therapy to transthyretin stabilizers. Similarly, Olatunji et al. [30] highlighted that both vutrisiran and eplontersen effectively reduce circulating transthyretin levels through gene-silencing mechanisms while demonstrating encouraging efficacy and acceptable safety profiles. Although the review by Olatunji et al. primarily focuses on hereditary transthyretin amyloidosis with polyneuropathy, the shared mechanism of transthyretin suppression suggests that these agents may have an expanding role in cardiac amyloidosis as evidence continues to emerge.

Olatunji et al. [30] proposed that future studies should investigate combination treatment strategies involving RNA-silencing therapies and transthyretin stabilizers such as tafamidis or acoramidis. Because these therapies target different stages of the disease process, combining transthyretin stabilization with suppression of transthyretin production may provide additive or synergistic clinical benefits. Nevertheless, no clinical studies have yet established the efficacy or safety of such combination approaches, and their role in routine clinical practice remains to be determined.

The rapidly evolving therapeutic landscape also raises several important clinical questions regarding optimal treatment selection, sequencing, timing of therapy initiation, and patient selection [28]. At present, the lack of direct comparative trials between tafamidis, acoramidis, vutrisiran, and eplontersen precludes robust comparisons of their relative efficacy. Future research should therefore prioritize head-to-head comparative effectiveness studies, evaluate the role of combination therapy, and identify patient subgroups most likely to benefit from each therapeutic strategy. Such studies will be essential for defining future treatment algorithms and optimizing individualized management of patients with ATTR-CM.

## 5. Conclusions

This systematic review suggests that tafamidis may contribute to clinical stabilization and improved survival outcomes in patients with ATTR-CM. Across observational and before–after studies, tafamidis treatment was associated with preservation of echocardiographic parameters and slower deterioration in functional status, while randomized trial evidence provides the strongest support for reduced mortality and clinical benefit. Although changes in cardiac biomarkers, including NT-proBNP and hs-cTnT, were heterogeneous and often not statistically significant, the overall evidence suggests that tafamidis primarily acts by slowing disease progression rather than reversing established myocardial damage. This lack of consistent biomarker improvement represents an important finding and suggests that NT-proBNP and hs-cTnT changes should not be used alone to define response to tafamidis.

Functional outcomes, including NYHA class, 6MWT, and KCCQ, may support the clinical benefit of tafamidis, particularly where randomized evidence is available. Mortality outcomes showed the most consistent signal in favor of tafamidis; however, observational findings should be interpreted cautiously because selection bias, limited comparability between treated and untreated cohorts, and possible immortal time bias may limit causal interpretation. In addition, available safety data indicate that tafamidis is generally well tolerated and does not appear to be associated with significant adverse effects.

Despite these encouraging findings, the evidence base remains limited by heterogeneity in study design, follow-up duration, outcome definitions, and representation of ATTR subtypes, particularly variant ATTR. Future large-scale prospective studies with standardized reporting of imaging, biomarker, functional, and safety outcomes are needed to better define the long-term impact of tafamidis therapy and optimize management strategies for patients with ATTR-CM.

## Figures and Tables

**Table 1:** PICO framework used to define the review question and outcomes of interest.

Patient/Problem/Population:	Adults with transthyretin cardiac amyloidosis
Intervention/Exposure	Tafamidis
Comparison	Placebo, no treatment initiation or change in baseline outcomes
Outcomes	**Echocardiographic Outcomes:** Global left ventricular strain (GLS), left ventricular ejection fraction (LVEF). **Cardiac Biomarker Outcomes:** N-terminal pro-B-type natriuretic peptide (NT-proBNP), high-sensitivity cardiac troponin T (hs-cTnT). **Functional Outcomes:** New York Heart Association (NYHA) Functional Classification, National Amyloidosis Centre (NAC) staging score, Kansas City Cardiomyopathy Questionnaire (KCCQ) score, 6-minute walk test (6MWT) score, all-cause mortality and safety.

**Table 2:** Study characteristics and ATTR subtype distribution among included studies.

			ATTR Type	
Study	Year	Sample Size	ATTRwt	Variant/Hereditary
Chamling et al. [10]	2023	40	40	0
Dobner et al. [19]	2024	91	88	3
Eguchi et al. [11]	2025	27	27	0
Falk et al. [20]	2021	72	67	5
Kuyama et al. [16]	2024	101	101	0
Maurer et al. [4]	2018	441	335	106
Ney et al. [17]	2024	62	62	0
Palmiero et al. [21]	2024	28	28	0
Rettl et al. [12]	2022	109	109	0
Rettl et al. [22]	2023	40	40	0
Shahi et al. [23]	2024	139	131	8
Shin et al. [15]	2026	77	17	60
Takashio et al. [13]	2023	180	180	0
Gawor-Prokopczyk et al. [24]	2025	115	94	21
Duca et al. [25]	2026	54	48	6
Zlibut et al. [14]	2026	56	56	0
Kuyama et al. [18]	2025	258	N/A ^1^	N/A

1: Not Applicable.

**Table 3:** Availability of outcome measures in the included studies.

Study	Year	Outcomes									
		LVEF	GLS	NT-proBNP	hs-cTnT	KCCQ	6MWT	NAC Score	NYHA Class	All-Cause Mortality	Safety
Chamling et al. [10]	2023	✓	✓	✓	✗	✗	✗	✓	✓	✓	✓
Dobner et al. [19]	2024	✓	✓	✓	✓	✗	✗	✓	✓	✓	✗
Eguchi et al. [11]	2025	✓	✓	✗	✗	✗	✗	✗	✓	✗	✗
Falk et al. [20]	2021	✗	✗	✓	✓	✗	✗	✗	✗	✓	✗
Kuyama et al. [16]	2024	✓	✓	✓	✓	✗	✗	✗	✗	✗	✗
Maurer et al. [4]	2018	✗	✗	✗	✗	✓	✓	✗	✓	✓	✓
Ney et al. [17]	2024	✓	✓	✓	✓	✗	✗	✓	✓	✗	✗
Palmiero et al. [21]	2024	✓	✓	✓	✓	✓	✓	✓	✓	✗	✗
Rettl et al. [12]	2022	✓	✓	✓	✓	✗	✓	✓	✓	✓	✗
Rettl et al. [22]	2023	✓	✓	✓	✓	✗	✓	✗	✓	✓	✗
Shahi et al. [23]	2024	✗	✗	✗	✗	✗	✗	✗	✗	✓	✗
Shin et al. [15]	2026	✓	✓	✓	✗	✗	✗	✓	✗	✓	✓
Takashio et al. [13]	2023	✓	✓	✓	✓	✗	✗	✗	✓	✓	✓
Gawor-Prokopczyk et al. [24]	2025	✗	✗	✓	✓	✗	✗	✗	✗	✓	✗
Duca et al. [25]	2026	✓	✓	✓	✓	✗	✓	✓	✓	✓	✗
Zlibut et al. [14]	2026	✓	✓	✓	✓	✗	✗	✓	✓	✓	✓
Kuyama et al. [18]	2025	✓	✓	✓	✓	✗	✗	✗	✓	✓	✗

**Table 4:** The different hospitalization endpoints as defined in each applicable study.

Study	Hospitalization Endpoint
Maurer et al. [4]	Cardiovascular-related hospitalization
Takashio et al. [13]	Hospitalization for worsening heart failure
Shin et al. [15]	Hospitalization for worsening heart failure
Gawor-Prokopczyk et al. [24]	Composite clinical endpoints as well as heart failure and transplant hospitalizations
Kuyama et al. [18]	Unplanned hospitalization for heart failure
Duca et al. [25]	Composite endpoint of all-cause mortality, cardiac transplantation, or heart failure hospitalization
Shahi et al. [23]	Cardiovascular-related hospitalization
Palmiero et al. [21]	Cardiovascular- and heart failure-related hospitalizations
Ney et al. [17]	Hospitalization for worsening heart failure
Kuyama et al. [16]	Composite endpoint of all-cause mortality and heart failure hospitalization
Dobner et al. [19]	Hospitalization for worsening heart failure

**Table 5:** Summary of evidence certainty across echocardiographic, biomarker, and functional outcomes using the GRADE framework.

Outcome	Certainty
GLS	Low
LVEF	Low
NT-proBNP	Low
hs-cTnT	Low
NYHA Class	Low to Moderate
NAC Staging	Low to Moderate
KCCQ	Low to Moderate
6MWT	Low to Moderate
All-Cause Mortality	Moderate
Safety	Low

## Data Availability

The original contributions presented in this study are included in the article/Supplementary Material. Further inquiries can be directed to the corresponding authors.

## Supplementary Materials

The following supporting information can be downloaded at https://www.mdpi.com/article/10.3390/jcm15155832/s1: File S1: PRISMA Checklist 2020; File S2: PRISMA 2020 for Abstract Checklist.

## Abbreviations

The following abbreviations are used in this manuscript:

ATTR	Transthyretin amyloidosis
ATTR-CM	Transthyretin cardiomyopathy
GLS	Global longitudinal strain
KCCQ	Kansas City Cardiomyopathy Questionnaire
6MWT	6-Minute Walk Test
NYHA	New York Heart Association
LVEF	Left ventricular ejection fraction
CA	Cardiac amyloidosis
NAC	National Amyloidosis Centre
RCT	Randomized controlled trial
NT-proBNP	N-terminal pro-B-type natriuretic peptide
ATTRwt	Wild-type transthyretin amyloidosis

## References

[1] Movila D.E., Motofelea A.C., Cozma D., Albai O., Sima A.C., Andor M., Ciocarlie T., Dragan S.R. (2025). Cardiac Amyloidosis: A Narrative Review of Diagnostic Advances and Emerging Therapies. Biomedicines.

[2] Ogieuhi I.J., Ugiomoh O.M.-A., Muzofa K., Callender K., Ayodeji J.D., Nnekachi N.P., Thiyagarajan B., Uduigwome E.O., Kapoor A., Odoeke M.C. (2024). Tafamidis therapy in transthyretin amyloid cardiomyopathy: A narrative review from clinical trials and real-world evidence. Egypt. Heart J..

[3] Bukhari S., Hamza M., Malik A. (2025). Transthyretin Cardiac Amyloidosis and Heart Failure: State-of-the-Art Review and Practice Guidance. Rev. Cardiovasc. Med..

[4] Maurer M.S., Schwartz J.H., Gundapaneni B., Elliott P.M., Merlini G., Waddington-Cruz M., Kristen A.V., Grogan M., Witteles R., Damy T. (2018). Tafamidis Treatment for Patients with Transthyretin Amyloid Cardiomyopathy. N. Engl. J. Med..

[5] Attal S., Kemner J., Alvir J., Barth S., Schuessler S. (2024). Tafamidis 61 mg Patient Characteristics and Persistency? A Retrospective Analysis of German Statutory Health Insurance Data (IQVIA^TM^ LRx). Cardiol. Ther..

[6] Page M.J., McKenzie J.E., Bossuyt P.M., Boutron I., Hoffmann T.C., Mulrow C.D., Shamseer L., Tetzlaff J.M., Akl E.A., Brennan S.E. (2021). The PRISMA 2020 statement: An updated guideline for reporting systematic reviews. BMJ.

[7] Wells G., O’Connell D., Peterson J., Welch M., Losos M., Tugwell P. (2020). The Newcastle–Ottawa Scale (NOS) for Assessing the Quality of Nonrandomized Studies in Meta-Analyses.

[8] Sahu V. (2026). Critiplot: A Critical Appraisal Plot Visualiser for Risk of Bias in Systematic Reviews and Meta-Analyses, Version v2.1.1.

[9] Sterne J.A.C., Savović J., Page M.J., Elbers R.G., Blencowe N.S., Boutron I., Cates C.J., Cheng H.-Y., Corbett M.S., Eldridge S.M. (2019). RoB 2: A revised tool for assessing risk of bias in randomised trials. BMJ.

[10] Chamling B., Bietenbeck M., Korthals D., Drakos S., Vehof V., Stalling P., Meier C., Yilmaz A. (2023). Therapeutic value of tafamidis in patients with wild-type transthyretin amyloidosis (ATTRwt) with cardiomyopathy based on cardiovascular magnetic resonance (CMR) imaging. Clin. Res. Cardiol..

[11] Eguchi C., Kawano H., Nishizawa R.H., Yoshimuta T., Ohno C., Kojima S., Minami T., Sato D., Eguchi M., Okano S. (2025). Comparing Effects of Tafamidis in Controlling Left Ventricular and Left Atrial Strains in Patients with Wild-Type Transthyretin Amyloid Cardiomyopathy. Circ. Rep..

[12] Rettl R., Duca F., Binder C., Dachs T.-M., Cherouny B., Camuz Ligios L., Mann C., Schrutka L., Dalos D., Charwat-Resl S. (2022). Impact of tafamidis on myocardial strain in transthyretin amyloid cardiomyopathy. Amyloid.

[13] Takashio S., Morioka M., Ishii M., Morikawa K., Hirakawa K., Hanatani S., Oike F., Usuku H., Kidoh M., Oda S. (2023). Clinical Characteristics, Outcome, and Therapeutic Effect of Tafamidis in Wild-Type Transthyretin Amyloid Cardiomyopathy. ESC Heart Fail..

[14] Zlibut A., Bietenbeck M., Akyol N., Vehof V., Bouras R., Isgandarova K., Meier C., Theofanidou M., Stalling P., Yilmaz A. (2026). CMR-based assessment of long-term effects of tafamidis in patients with cardiac transthyretin amyloidosis. Clin. Res. Cardiol..

[15] Shin H., Jeon K., Kim D., Hong D., Park M., Yoon S.E., Kim S.J., Kim J.-S., Kim K., Choi J.-O. (2026). Real-World Efficacy of Tafamidis in Korean ATTR-CM Patients: A Retrospective Observational Strain Analysis. Korean Circ. J..

[16] Kuyama N., Takashio S., Oguni T., Yamamoto M., Hirakawa K., Ishii M., Hanatani S., Oda S., Matsuzawa Y., Usuku H. (2024). Cardiac Biomarker Change at 1 Year After Tafamidis Treatment and Clinical Outcomes in Patients with Transthyretin Amyloid Cardiomyopathy. J. Am. Heart Assoc..

[17] Ney S., Gertz R.J., Pennig L., Nies R.J., Holtick U., Völker L.A., Wunderlich G., Seuthe K., Hohmann C., Metze C. (2024). Multiparametric Monitoring of Disease Progression in Contemporary Patients with Wild-Type Transthyretin Amyloid Cardiomyopathy Initiating Tafamidis Treatment. J. Clin. Med..

[18] Kuyama N., Izumiya Y., Takashio S., Usuku H., Tabira A., Oguni T., Yamamoto M., Hirakawa K., Ishii M., Tabata N. (2025). Long-Term Effect of Tafamidis on Clinical Parameters and Prognostic Predictors in Patients with Transthyretin Amyloid Cardiomyopathy. Circ. J..

[19] Dobner S., Zarro S., Wieser F., Kassar M., Alaour B., Wiedemann S., Bakula A., Caobelli F., Stortecky S., Gräni C. (2024). Effect of Timely Availability of TTR-Stabilizing Therapy on Diagnosis, Therapy, and Clinical Outcomes in ATTR-CM. J. Clin. Med..

[20] Falk R.H., Haddad M., Walker C.R., Dorbala S., Cuddy S.A.M. (2021). Effect of Tafamidis on Serum Transthyretin Levels in Non-Trial Patients with Transthyretin Amyloid Cardiomyopathy. J. Am. Coll. Cardiol. CardioOnc..

[21] Palmiero G., Monda E., Verrillo F., Dongiglio F., Cirillo C., Caiazza M., Rubino M., Cirillo A., Fusco A., Diana G. (2024). Impact of Tafamidis on Delaying Clinical, Functional, and Structural Cardiac Changes in Patients with Wild-Type Transthyretin Amyloid Cardiomyopathy. J. Clin. Med..

[22] Rettl R., Wollenweber T., Duca F., Binder C., Cherouny B., Dachs T.-M., Camuz Ligios L., Schrutka L., Dalos D., Beitzke D. (2023). Monitoring tafamidis treatment with quantitative SPECT/CT in transthyretin amyloid cardiomyopathy. Eur. Heart J.—Cardiovasc. Imaging.

[23] Shahi K., Miller R.J.H., Dykstra S., Feng Y., Howlett J.G., Jimenez-Zepeda V., Veenhuyzen J., White J.A., Fine N.M. (2024). Baseline Predictors of Adverse Outcomes for Transthyretin Amyloidosis Cardiomyopathy Patients Treated and Untreated with Tafamidis: A Canadian Referral Center Experience. J. Clin. Med..

[24] Gawor-Prokopczyk M., Grzybowski J., Lipowska M., Truszkowska G., Ponińska J., Rajtar-Salwa R., Susuł M., Keller D., Straburzyńska-Migaj E., Dąbrowska-Kugacka A. (2026). Multicenter study of patients with transthyretin cardiac amyloidosis in Poland: Clinical characteristics, genotyping, electrocardiographic features, outcome predictors, and tafamidis treatment outcomes. Pol. Heart J..

[25] Duca F., Poledniczek M., Kronberger C., Binder C., Rettl R., Camuz-Ligios L., Agis H., Koschutnik M., Donà C., Badr-Eslam R. (2025). Serial extracellular volume quantification using cardiac magnetic resonance imaging in transthyretin amyloidosis patients treated with tafamidis. Eur. Radiol..

[26] Kittleson M.M., Maurer M.S., Ambardekar A.V., Bullock-Palmer R.P., Chang P.P., Eisen H.J., Nair A.P., Nativi-Nicolau J., Ruberg F.L., On behalf of the American Heart Association Heart Failure and Transplantation Committee of the Council on Clinical Cardiology (2020). Cardiac Amyloidosis: Evolving Diagnosis and Management: A Scientific Statement from the American Heart Association. Circulation.

[27] Griffin J.M., Grodin J.L., Ruberg F.L., Masri A., Hanna M., Maurer M.S. (2025). Current Landscape of Therapies for Transthyretin Amyloid Cardiomyopathy. J. Am. Coll. Cardiol. Heart Fail..

[28] Dimza M., Vasilakis G., Grodin J.L. (2025). Transthyretin Amyloid Cardiomyopathy: The Plot Thickens as Novel Therapies Emerge. US Cardiol. Rev..

[29] Margolin E., Stern L.K., Argiro A., Rosenthal J.L., Urey M.A., Alexander K.M. (2025). Current and Future Treatment Landscape of Transthyretin Amyloid Cardiomyopathy. Cardiol. Ther..

[30] Olatunji G., Kokori E., Abraham I.C., Omoworare O., Olatunji D., Ezeano C., Emmanuel Adeoba B., Stanley A.C., Oluwatobiloba A.M., Oluwademilade O.B. (2024). A mini-review of Vutrisiran and Eplontersen in hereditary transthyretin-mediated amyloidosis with polyneuropathy. Medicine.

